# Pedicled omental flap construction: A useful adjunct to completion proctectomy or pouch excision for inflammatory bowel disease

**DOI:** 10.1007/s10151-026-03305-9

**Published:** 2026-05-03

**Authors:** S. D. Holubar, J. Pangrace, F. Dermuth, C. Prien, H. Kessler

**Affiliations:** Department of Colon & Rectal Surgery, Cleveland Clinic Lerner College of Medicine and Case Western Reserve University, Cleveland Clinic Main Campus, 9500 Euclid Ave, A30, Cleveland, OH 44122 USA

Inflammatory bowel disease patients often require a completion proctectomy or pouch excision, typically via intersphincteric dissection [1–3]. Unfortunately, up to 30% of patients will have non-healed wounds at 1 year [4, 5]. The TOpClass (Treatment Optimization and Classification) Consortium for perianal Crohn’s disease (pCD) recently classified these wounds as Class 4 pCD [6, 7]. Prophylactic intra-abdominal omental pedicled flaps have been shown to improve perineal wound healing rates [8–12]. Pedicled omental flap construction (Fig. 1) involves high ligation of one of the gastroepiploic (GE) arteries and short gastric arteries, taking care to preserve the GE trunk, which then perfuses the flap via the preserved contralateral GE artery (Fig. 2 and 3).

**Fig. 1:**
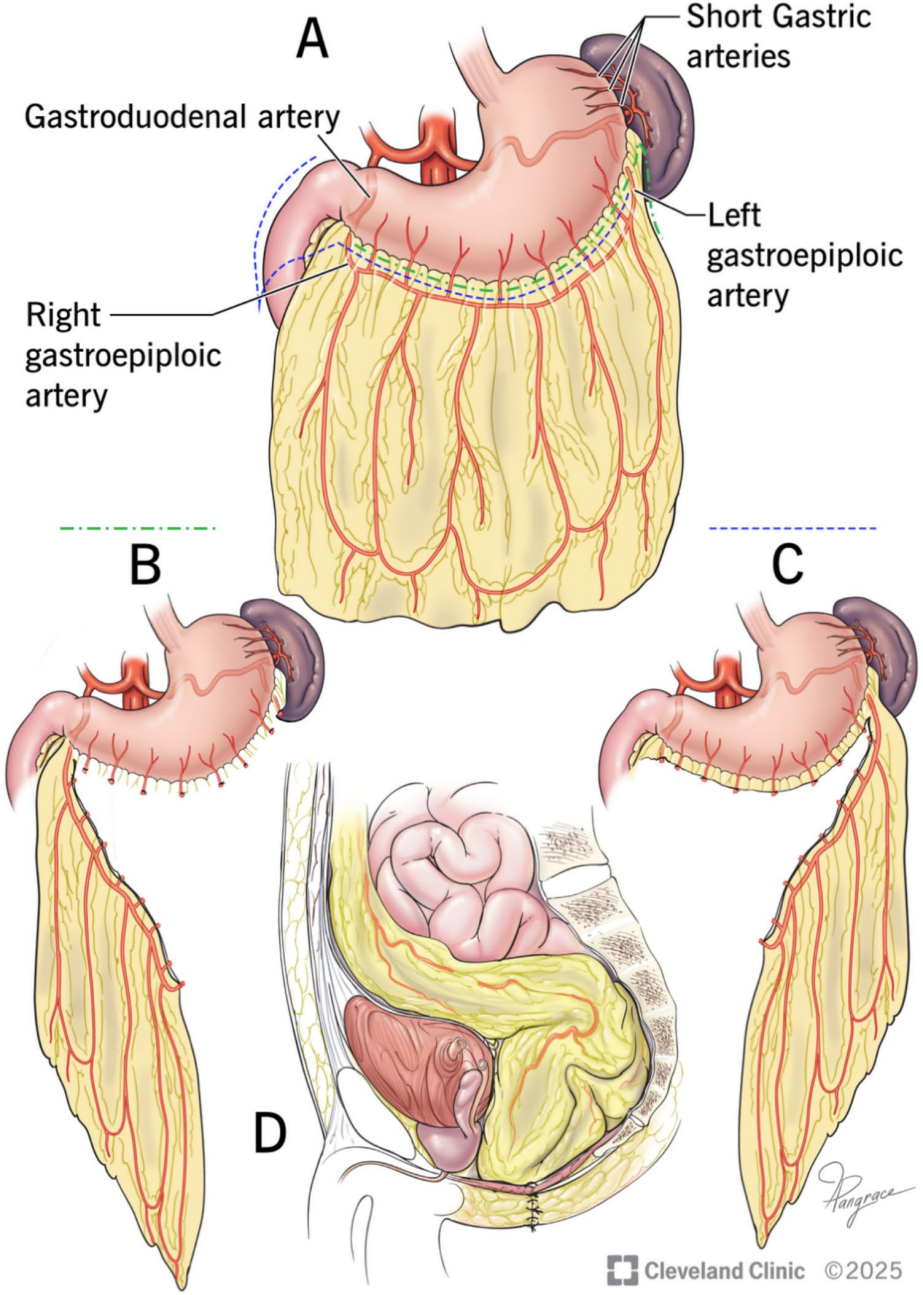
Construction of a pedicled omental flap based on one gastroepiploic (GE) artery. The nascent flap (**A**) is based on the right (**B**) or left (**C**) GE artery after high ligation of the contralateral GE artery and short gastric arteries close to the gastric body, taking great care to preserve the GE trunk, which then supplies the flap. The flap is then passed in the left or right paracolic gutter to fill the dead space of the pelvis (**D**) after the intersphincteric dissection has been completed and the perineal defect closed (bottom panel **D**). © Cleveland Clinic Foundation 2025. GE, gastroepiploic

**Fig. 2:**
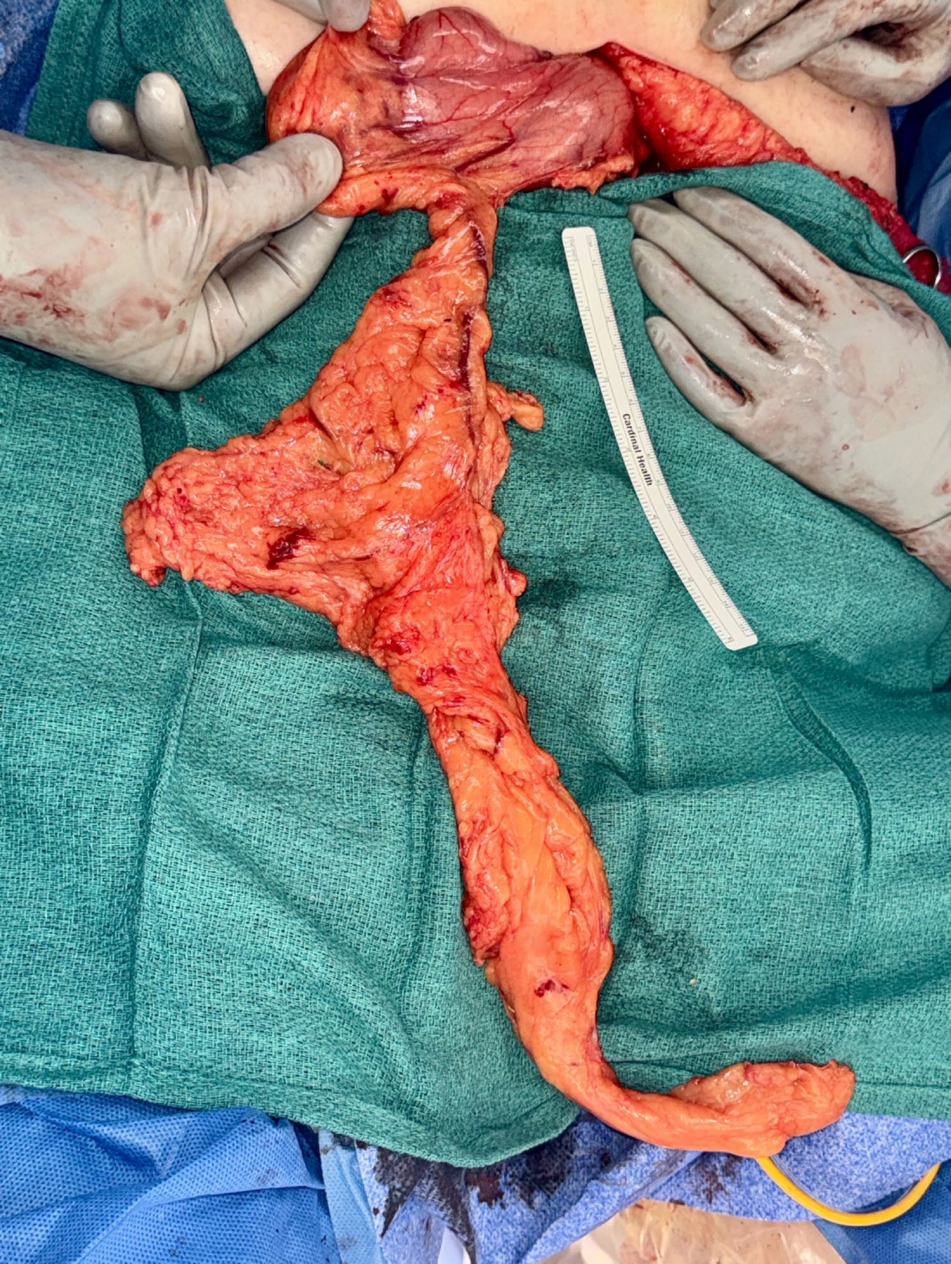
In situ appearance of an omental pedicled flap based on the right gastroepiploic (GE) artery in a patient with prior subtotal omentectomy undergoing pouch excision. The residual wad of omentum high in the left upper quadrant was carefully mobilized from attachments to the spleen and gastric fundus and a high ligation of the left GE artery and short gastric performed, taking great care to preserve the main GE trunk. A nasogastric tube may be placed intraoperatively to manageme anticipated gastric ileus. © Cleveland Clinic Foundation, 2025. GE, gastroepiploic

**Fig. 3:**
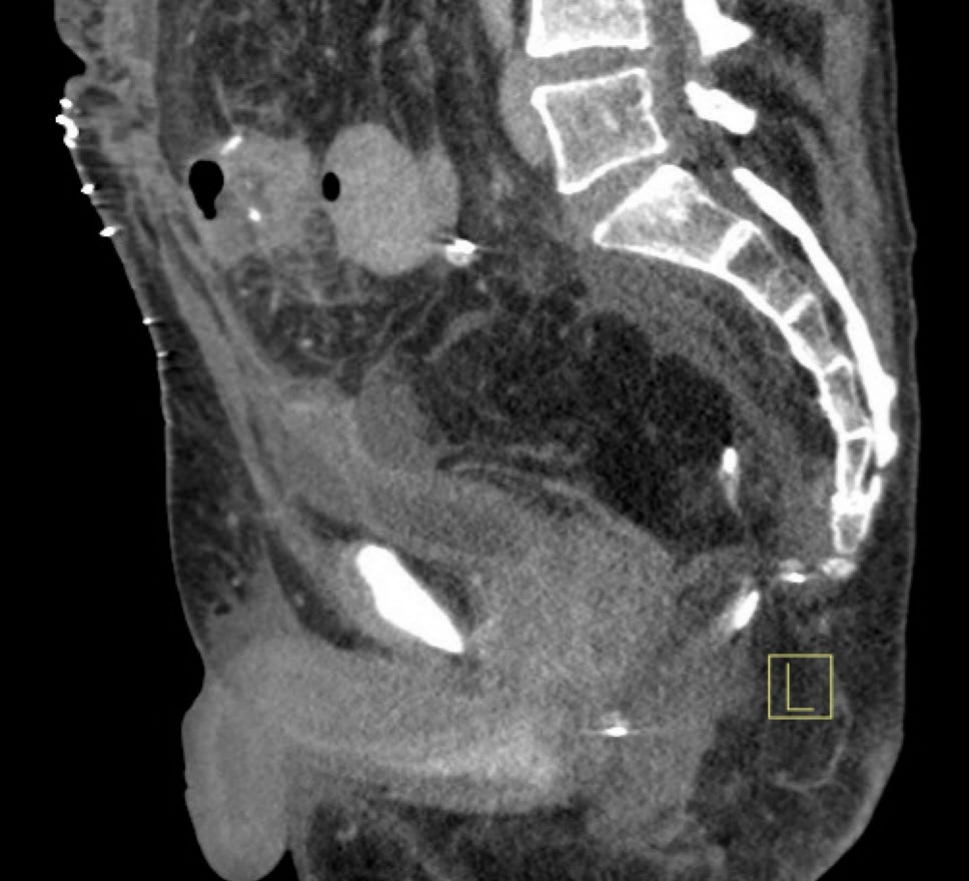
Postoperative computed tomography (CT) appearance of an omental pedicled flap filling the pelvis after pouch excision. Note the partially visible pelvic closed-suction drain, still in situ, positioned in the deepest portion of the pelvis adjacent to the closure of the levators and perineal defect. © Cleveland Clinic Foundation 2025. CT, computed tomography

## Data Availability

The data used in this study are not publicly available but can be made available upon reasonable request.
